# Clinical DVH metrics as a loss function for 3D dose prediction in head and neck radiotherapy

**DOI:** 10.1007/s11548-026-03761-6

**Published:** 2026-08-13

**Authors:** Ruochen Gao, Marius Staring, Frank Dankers

**Affiliations:** 1 Division of Image Processing, Department of Radiology, Leiden University Medical Center, Leiden, The Netherlands; 2 Department of Radiation Oncology, Leiden University Medical Center, Leiden, The Netherlands

**Keywords:** Dose prediction, Deep learning, DVH metric loss, Bit-mask

## Abstract

**Purpose:**

Deep learning-based three-dimensional (3D) dose prediction is widely used in automated radiotherapy workflows. However, most existing models are trained with voxel-wise regression losses, which are poorly aligned with clinical plan evaluation criteria based on dose–volume histogram (DVH) metrics. This study aims to develop a clinically guided loss formulation that directly optimizes clinically used DVH metrics while remaining computationally efficient for head and neck (H&N) dose prediction.

**Methods:**

We propose a clinical DVH metric loss (CDM loss) that incorporates differentiable *D-metrics* and surrogate *V-metrics*, together with a lossless bit-mask region-of-interest (ROI) encoding to improve training efficiency. The method was evaluated on 174 H&N patients using a temporal split (137 training, 37 testing).

**Results:**

Compared with MAE- and DVH curve-based losses, CDM loss substantially improved target coverage and satisfied all clinical constraints. Using a standard 3D U-Net, the PTV Score was reduced from 1.544 (MAE) to 0.491 (MAE + CDM), while OAR sparing remained comparable. Bit-mask encoding reduced training time by 82.2% and lowered GPU memory usage.

**Conclusion:**

Directly optimizing clinically used DVH metrics enables 3D dose predictions that are better aligned with clinical treatment planning criteria than conventional voxel-wise or DVH curve-based supervision. The proposed CDM loss, combined with efficient ROI bit-mask encoding, provides a practical and scalable framework for H&N dose prediction.

**Supplementary Information:**

The online version contains supplementary material available at https://doi.org/10.1007/s11548-026-03761-6.

## Introduction

Radiotherapy (RT) is widely used in cancer treatment to deliver sufficient radiation to eradicate tumor cells while protecting surrounding healthy tissues. Conventional treatment planning directly optimizes beam parameters within the treatment planning system (TPS), often requiring multiple rounds of manual refinements and substantial planner expertise. To improve efficiency and consistency, modern workflows introduce dose prediction as an intermediate step, providing an anatomy-informed estimate of a clinically realistic three-dimensional (3D) dose distribution. This predicted dose can then be used to guide subsequent TPS optimization to generate deliverable plan [1]. Among all treatment sites, head and neck (H&N) radiotherapy is particularly challenging due to its intricate anatomy, a large number of organs at risk (OARs), and steep dose gradients between planning target volumes (PTVs) and nearby healthy organs [2]. These complexities make high-quality optimization particularly demanding and contribute to substantial inter-planner and inter-institutional variability in plan quality [3]. Therefore, developing accurate and automated 3D dose prediction models tailored for H&N treatment is essential to reduce planning workload and promote standardization in clinical practice.

**Fig. 1:**
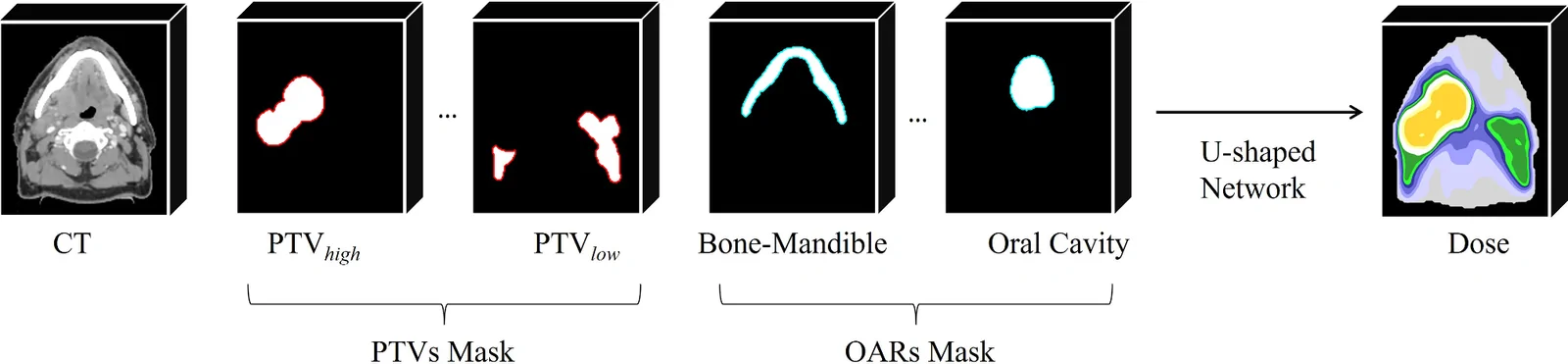
Workflow of a typical deep learning-based H&N dose prediction method

Recently, deep learning (DL) methods have shown strong potential for learning complex mappings between patient anatomy and clinical dose patterns. Most DL-based methods use 3D convolutional or transformer-based U-shaped architectures [4–7], taking CT and region-of-interest (ROI) masks as input to generate 3D voxel-wise dose predictions, as shown in Fig. 1. These approaches generally achieve high voxel-wise accuracy by using regression losses such as mean absolute error (MAE) or mean squared error (MSE). However, voxel-wise accuracy is not the primary criterion used in clinical evaluation. Instead, clinicians focus on achieving adequate PTV coverage and sufficient OAR sparing, which are typically assessed using dose–volume histogram (DVH)-derived metrics. Since standard regression losses do not directly optimize these clinical objectives, DL-based models may achieve high numerical accuracy, yet still perform sub-optimally from a clinical standpoint.

To bridge this gap, several studies have incorporated differentiable DVH curve losses, which allow comparison of predicted and ground truth DVH curves during training [8–11]. However, in clinical practice, plan evaluation relies on a set of *D-* and *V-metrics* rather than full DVH curves (Table 1). While *D-metrics* are differentiable and can be incorporated into training [10, 11], *V-metrics* contain threshold operations that are inherently non-differentiable, posing challenges for the gradient-based training framework. Therefore, effective methods for directly optimizing *V-metrics* remain limited, and a comprehensive framework that jointly supports both *D-* and *V-metrics* is still needed.

**Table 1:** Definitions of common *D-* and *V-metrics* in H&N RT

Parameter	Definition
$D_{x\%}$	Minimum dose received by the hottest *x*% of the ROI volume
$D_{x~\text {cc}}$	Minimum dose received by the hottest *x* cc of the ROI
$D_{\text {mean}}$	Mean dose within the ROI
$V_{x\%}$	Volume percentage of an ROI receiving at least *x*% of the prescription dose
$V_{x~\text {Gy}}$	Volume percentage of an ROI receiving at least *x* Gy

Another practical challenge arises from the large number of OARs in H&N anatomy. Dose prediction networks typically require the full 3D volume as input to guarantee spatial dose continuity. Current methods generally implement these ROIs as one input channel per ROI. As a result, when many OARs are included, the number of input channels grows rapidly, leading to substantial preprocessing overhead, higher CPU–to–GPU data transfer cost, and longer training time. Although merging all ROIs into a single indexed mask could reduce input dimensionality, this approach faces two major issues: (1) Many OARs overlap spatially (e.g., the brain and brainstem overlap), making direct merging impossible, and (2) the numerical labels introduce unintended ordinal bias (e.g., a label “9” may be treated differently from a label “1” purely because it is larger), even though ROI labels should carry no numeric significance. As a result, prior studies often limit the input to a subset of OARs [5–7, 10–12], which is restrictive, as clinical evaluation typically involves a much broader set of OARs.

To address these limitations, we propose a clinical DVH metric loss (CDM loss) together with an efficient bit-mask ROI encoding strategy for 3D H&N dose prediction, enabling direct optimization of clinically defined dose evaluation metrics while maintaining computational efficiency. Our main contributions are summarized as follows:

- We introduce a clinical DVH metric loss (CDM loss) that combines differentiable formulations for *D-metrics* with differentiable approximations for *V-metrics*. By directly optimizing the metrics used in clinical plan evaluation, we show that even a standard 3D U-Net can achieve clinically satisfactory dose predictions.
- We develop an efficient lossless bit-mask encoding for ROI representation that reduces CPU preprocessing, CPU–to–GPU data transfer, and GPU memory usage through lightweight, on-demand decoding. This strategy substantially accelerates training, while supporting the full set of OARs required for clinical evaluation.
- We organize the clinical evaluation dose metrics used by CDM loss function through a simple JSON-based template, as summarized in Table 2. This template specifies the ROIs and their associated dose metrics in a clear, structured, and easily updated format, ensuring that the training objectives remain aligned with clinical evaluation criteria used in a given institute.

**Table 2:** H&N RT clinical plan evaluation template used in our institution. The PTVs are denoted by their prescribed dose levels (e.g., $\text {PTV}_{70}$ for 70 Gy). ROIs with “L/R” denote paired organs and are evaluated independently for the left and right sides. “Aim” represents the desired planning goal, while “Constraint” denotes a mandatory criterion. The template is implemented via a JSON file

ROI	Metric	Aim	Constraint
$\text {PTV}_{54.25}$	$V_{95\%}$		$\ge 98\%$
$\text {PTV}_{54.25}$	$D_{\text {mean}}$	$\le 102\%$	
$\text {PTV}_{70}$	$V_{95\%}$		$\ge 98\%$
$\text {PTV}_{70}$	$D_{0.03\text {cc}}$	$\le 107\%$	$\le 110\%$
$\text {PTV}_{70}$	$D_{\text {mean}}$	$\le 102\%$	
SpinalCord	$D_{0.03\text {cc}}$		$\le 50\,\text {Gy}$
SpinalCord + 3 mm	$D_{0.03\text {cc}}$		$\le 52\,\text {Gy}$
Brainstem_Surf	$D_{0.03\text {cc}}$		$\le 60\,\text {Gy}$
Brainstem_Core	$D_{0.03\text {cc}}$		$\le 54\,\text {Gy}$
Brain	$D_{0.03\text {cc}}$	$\le 65\,\text {Gy}$	
Brain	$D_{2\%}$	$\le 70\,\text {Gy}$	
Cochlea_(L/R)	$D_{\text {mean}}$	$\le 45\,\text {Gy}$	
Parotid_(L/R)	$D_{\text {mean}}$	$\le 28\,\text {Gy}$	
Glnd_Submand_(L/R)	$D_{\text {mean}}$	$\le 35\,\text {Gy}$	
Oral_Cavity	$D_{\text {mean}}$	$\le 28\,\text {Gy}$	
Musc_Constrict_S	$D_{\text {mean}}$	$\le 40\,\text {Gy}$	
Musc_Constrict_M	$D_{\text {mean}}$	$\le 40\,\text {Gy}$	
Musc_Constrict_I	$D_{\text {mean}}$	$\le 40\,\text {Gy}$	
Cricopharyngeus	$D_{\text {mean}}$	$\le 40\,\text {Gy}$	
Larynx_SG	$D_{\text {mean}}$	$\le 40\,\text {Gy}$	
Glottic_Area	$D_{\text {mean}}$	$\le 40\,\text {Gy}$	
Bone_Mandible	$D_{2\%}$	$\le 70\,\text {Gy}$	
Bone_Mandible-PTV	$D_{2\%}$	$\le 50\,\text {Gy}$	
Eye_(L/R)	$D_{0.03\text {cc}}$	$\le 35\,\text {Gy}$	
Lens_(L/R)	$D_{0.03\text {cc}}$	$\le 6\,\text {Gy}$	
Pituitary	$D_{\text {mean}}$	$\le 20\,\text {Gy}$	
OpticNrv_(L/R)	$D_{0.03\text {cc}}$	$\le 55\,\text {Gy}$	

## Materials

### Dataset

In this study, data were collected retrospectively from 174 H&N cancer patients treated at Leiden University Medical Center from February 2021 to July 2025. The cohort covered a range of tumor sites, including the oropharynx, hypopharynx, nasopharynx, larynx, and oral cavity. All patients were treated using 6-MV dual-arc, full-rotation volumetric modulated arc therapy (VMAT).

To reflect real-world clinical application, the dataset was divided chronologically. Patients treated between February 2021 and July 2024 (n=137) were assigned to the training cohort, while those treated between August 2024 and July 2025 (n=37) formed the test cohort, enabling temporal (out-of-time) validation on later cases.

The original CT images had an average resolution of $1.2 \times 1.2 \times 2\,\textrm{mm}^3$, whereas the dose grid resolution was fixed at $2 \times 2 \times 2\,\textrm{mm}^3$. To ensure uniform spatial resolution, all CT scans were resampled to $2 \times 2 \times 2\,\textrm{mm}^3$. Cropping or padding was performed around the treatment isocenter to obtain a uniform volume size of (160, 192, 256).

### Evaluation metrics

To quantitatively evaluate model performance, three complementary evaluation metrics are defined: PTV Score, OAR Score, and Dose Score. The PTV Score and OAR Score are directly derived from the clinical plan evaluation template used in our institution (Table 2).

Let $\mathcal {P}$ denote the set of all PTVs, and $\mathcal {O}$ denote the set of all OARs. For each ROI $r \in \mathcal {P} \cup \mathcal {O}$, a corresponding set of clinical evaluation dose metrics $\mathcal {M}_r$ is defined according to Table 2. For example, $\mathcal {M}_r = \{V_{95\%},~D_{\text {mean}},~D_{0.03\text {cc}}\}$ for $\text {PTV}_{70}$, and $\mathcal {M}_r = \{D_{0.03\text {cc}},~D_{2\%}\}$ for Brain. For each metric $m \in \mathcal {M}_r$, let $M^{\text {pred}}_{r,m}$ and $M^{\text {gt}}_{r,m}$ denote the predicted and ground truth (clinical) values, respectively.

The PTV Score is defined as:

1 $$\begin{aligned} \text {PTV Score} = \frac{1}{|\mathcal {P}|} \sum _{p \in \mathcal {P}} \frac{1}{|\mathcal {M}_p|} \sum _{m \in \mathcal {M}_p} \left| M^{\text {pred}}_{p,m} - M^{\text {gt}}_{p,m} \right| , \end{aligned}$$

where all PTV metrics are normalized to the prescribed dose and expressed in percentage (%). Similarly, the OAR Score is defined as:

2 $$\begin{aligned} \text {OAR Score} = \frac{1}{|\mathcal {O}|} \sum _{o \in \mathcal {O}} \frac{1}{|\mathcal {M}_o|} \sum _{m \in \mathcal {M}_o} \left| M^{\text {pred}}_{o,m} - M^{\text {gt}}_{o,m} \right| , \end{aligned}$$

where all OAR metrics are measured in absolute dose (Gy).

The Dose Score is also reported to quantify the overall voxel-wise difference. Let Ω denote the set of all voxels within the dose grid, and $D^{\text {pred}}(v)$ and $D^{\text {gt}}(v)$ be the predicted and clinical dose at voxel $v \in \Omega$. The Dose Score, reported in Gy, is defined as:

3 $$\begin{aligned} \text {Dose Score} = \frac{1}{|\Omega |} \sum _{v \in \Omega } \left| D^{\text {pred}}(v) - D^{\text {gt}}(v) \right| . \end{aligned}$$

## Methods

### CDM loss formulation

Based on the clinical plan evaluation criteria summarized in Table 2, we formulate the proposed CDM loss that directly optimizes the specified dose metrics. It incorporates both *D-* and *V-metrics*, allowing end-to-end training guided by clinical evaluation criteria.

**Fig. 2:**
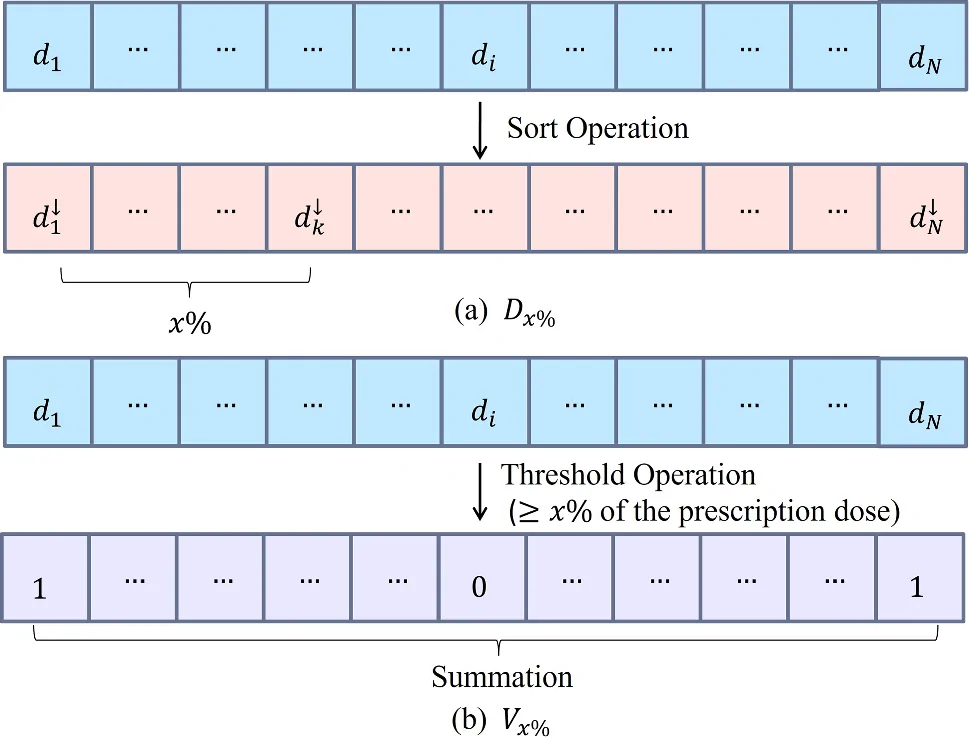
**a** *D-metrics* (e.g., $D_{x\%}$) are obtained by sorting voxel doses within an ROI and selecting the value corresponding to the hottest x% of the volume. **b** *V-metrics* (e.g., $V_{x\%}$) represent the fraction of ROI voxels receiving at least x% of the prescription dose

#### Differentiable formulation of *D-metrics*

As shown in Fig. 2a, given a set of voxel doses $\{d_i\}_{i=1}^{N}$ within an ROI of size *N*, sorted in descending order as $d^{\downarrow }_{1} \ge d^{\downarrow }_{2} \ge \dots \ge d^{\downarrow }_{N}$, the minimum dose received by the hottest x% of the ROI volume is denoted as:

4 $$\begin{aligned} D_{x\%} = d^{\downarrow }_{k}, ~~\text {where } k = \big \lceil \frac{x}{100}N \big \rceil . \end{aligned}$$

Thus, $D_{x\%}$ corresponds to the *k*-th largest dose value, which can be found at index *k* after sorting. Similarly, the minimum dose received by the hottest *x* cc of the ROI, denoted as $D_{x~\text {cc}}$, is computed in an analogous manner:

5 $$\begin{aligned} D_{x~\text {cc}} = d^{\downarrow }_{k}, ~~\text {where } k = \big \lceil \frac{x}{V_\text {voxel}} \big \rceil , \end{aligned}$$

where $V_\text {voxel}$ denotes the volume of a single voxel (in cc). Both $D_{x\%}$ and $D_{x~\text {cc}}$ can therefore be interpreted as quantile-based metrics, differing only in whether the cutoff is defined by a fractional or absolute volume.

Following prior work [10, 11], we use a top-*k* algorithm that computes the required quantiles without explicitly sorting all voxels, offering both differentiability and computational efficiency.

Finally, the mean dose $D_{\textrm{mean}} = \frac{1}{N} \sum _{i=1}^{N} d_i$ is inherently differentiable.

#### Differentiable surrogate of *V-metrics*

*V-metrics*, including $V_{x\%}$ and $V_{x~\text {Gy}}$, describe the fraction of an ROI receiving at least a specified dose level. As illustrated in Fig. 2b, $V_{x\%}$ denotes the fraction of the ROI receiving at least x% of the prescription dose. Given the prescription dose $D_{\text {presc}}$, the corresponding threshold is $T = x D_{\text {presc}} / 100$ Gy. The computation of $V_{x\%}$ is equivalent to counting the number of voxels with dose $d_i \ge T$, normalized by the total number of voxels *N*:

6 $$\begin{aligned} V_{x\%} = \frac{1}{N} \sum _{i=1}^{N} H(d_i - T), \end{aligned}$$

where H(·) is the non-differentiable Heaviside step function. The same principle applies to $V_{x~\text {Gy}}$, differing only in that the threshold is specified in absolute dose.

To enable gradient-based training, we approximate *H* with a logistic sigmoid $\sigma _{\alpha }(t) = 1/(1+e^{-\alpha t})$, with slope parameter α>0:

7 $$\begin{aligned} V_{x\%}^{\textrm{approx}} = \frac{1}{N} \sum _{i=1}^{N} \sigma _{\alpha }\left( d_i - T\right) . \end{aligned}$$

A larger α causes $\sigma _{\alpha }$ to more closely approximate the hard threshold, but may increase the risk of gradient saturation: When α is too large, most voxels have $|\alpha (d_i - T)| \gg 1$, placing them in the flat tails of the sigmoid where $\sigma '_\alpha (t) \approx 0$. Therefore, rather than arbitrarily increasing α, we seek the smallest value that ensures sufficient approximation accuracy while mitigating the risk of gradient saturation.

#### Selection of α via a bounded approximation error

In the differentiable surrogate of *V-metrics*, the sigmoid slope parameter α controls the trade-off between approximation accuracy of the hard threshold and gradient stability during training. Rather than choosing α heuristically, we determine it using an explicit bound on the approximation error. The full derivation is provided in Supplementary Material.

To ensure that the absolute approximation error of a *V-metric* does not exceed a user-defined tolerance ε, it suffices to choose α such that

8 $$\begin{aligned} \alpha \ge \frac{1}{m} \ln \!\left( \frac{1 - q_m}{\varepsilon - \tfrac{1}{2}q_m} \right) , \end{aligned}$$

where *m* denotes a dose margin (in Gy) around the dose threshold *T* and $q_m$ is the fraction of voxels in the ROI whose dose values satisfy $|d_i - T| \le m$. The parameter ε specifies the maximum tolerated absolute error of the *V-metric*.

In practice, $q_m$ is estimated from the training set, and the smallest α satisfying Eq. (8) is used to balance approximation accuracy and training stability.

#### Overall loss formulation

Using the differentiable formulation of *D-metrics* and the differentiable surrogate of *V-metrics* described above, we compute each metric in the template (Table 2) for both the predicted and ground truth dose in the same way. The loss is then defined as:

9 $$\begin{aligned} \mathcal {L}_{\textrm{CDM}} = \sum _{r \in \mathcal {R}} \sum _{k \in \mathcal {M}_r} w_{r,k}\, \big | M^{\textrm{pred}}_{r,k} - M^{\textrm{gt}}_{r,k} \big |, \end{aligned}$$

where $M^{\textrm{pred}}_{r,k}$ and $M^{\textrm{gt}}_{r,k}$ denote the predicted and ground truth metric *k* for ROI *r*, and $w_{r,k}$ denotes a weight. To preserve voxel-wise accuracy of the predicted dose distribution, we also use the MAE loss:

10 $$\begin{aligned} \mathcal {L}_{\textrm{MAE}} = \frac{1}{|\Omega |} \sum _{v \in \Omega } \big | D^{\textrm{pred}}(v) - D^{\textrm{gt}}(v) \big |. \end{aligned}$$

The final objective combines both components:

11 $$\begin{aligned} \mathcal {L}_{\textrm{total}} = \lambda _1 \mathcal {L}_{\textrm{MAE}} + \lambda _2 \mathcal {L}_{\textrm{CDM}}, \end{aligned}$$

where $\lambda _1$ and $\lambda _2$ balance voxel-wise consistency and alignment with clinical objectives.

**Fig. 3:**
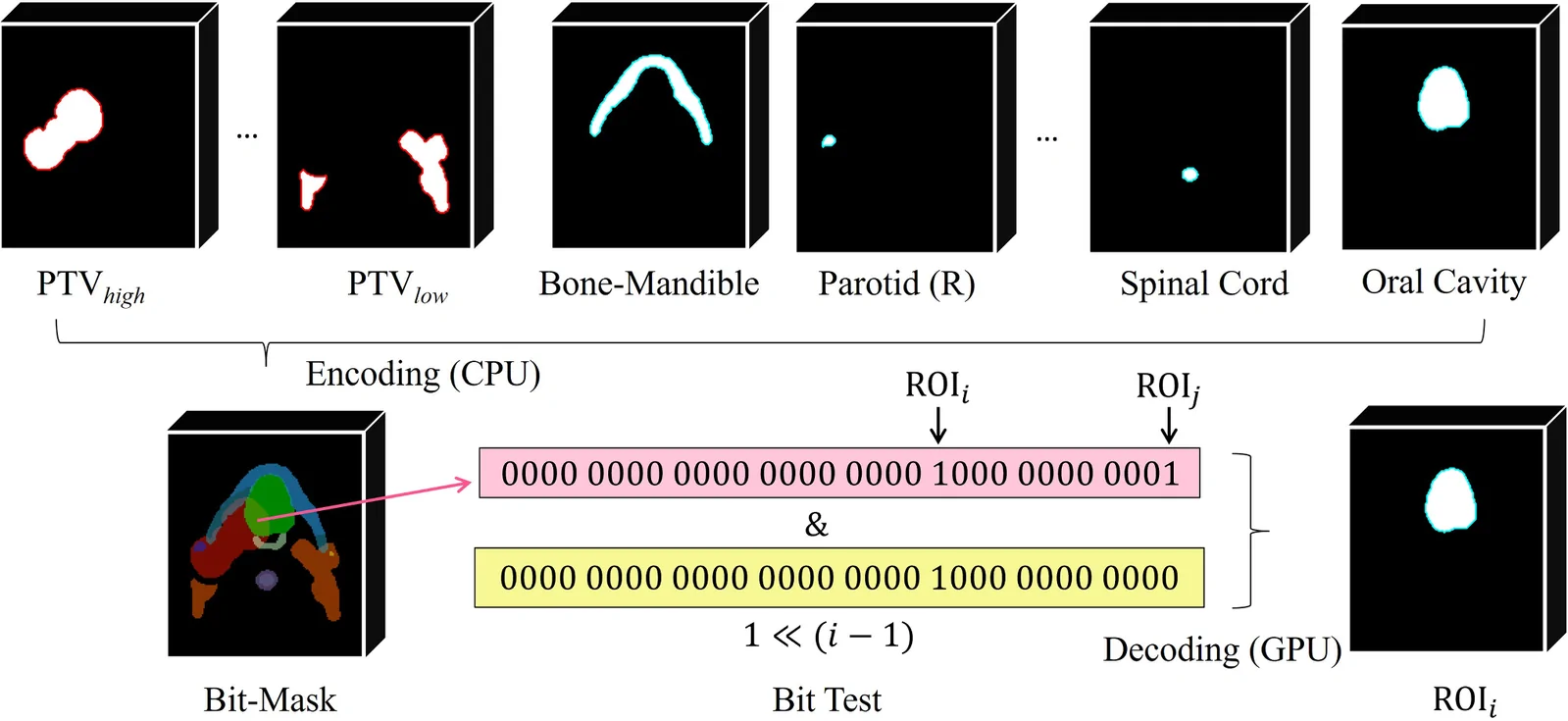
Multiple individual ROI masks are losslessly encoded into a single integer bit-mask, where each bit position represents a specific ROI (illustrated in pink). During decoding, ROI$_i$ is recovered by a GPU bitwise AND between the bit-mask and the single bit-mask (1≪(i-1)), illustrated in yellow

### Bit-mask encoding

The CDM loss in Section CDM loss formulation requires access to all ROIs listed in Table 2. In H&N RT, the number of ROIs is typically large (often exceeding twenty), and many of them overlap spatially. In conventional dose prediction pipelines, each ROI is stored as an independent 3D binary mask channel using a one-hot encoding. When full 3D volumes are used as network input, this representation leads to substantial overhead in CPU preprocessing and CPU–to–GPU data transfer.

To alleviate these inefficiencies, we introduce a lossless bit-mask encoding as a compact intermediate representation for ROI handling, as illustrated in Fig. 3. Formally, let $S_i(\textbf{r})\in \{0,1\}$ denote voxel membership in ROI$_i$. Assigning one bit position to each ROI yields the lossless encoding

12 $$\begin{aligned} B(\textbf{r}) = \sum _{i=1}^{N_{\textrm{ROI}}} S_i(\textbf{r})\, 2^{\,i-1}, \qquad N_{\textrm{ROI}} \le B_{\textrm{max}}, \end{aligned}$$

where $B_{\textrm{max}}$ denotes the bit width of the integer type. In this work, a 32-bit unsigned integer (uint32) is used, supporting up to 32 ROIs. Overlapping anatomical structures are represented by activating multiple bits within the same voxel, preserving all ROI relationships in a single channel.

A key advantage of this representation arises during data preprocessing. With conventional one-hot encoding, geometric augmentations must be applied independently to each ROI channel, causing preprocessing cost to scale with the number of ROIs, whereas bit-mask encoding allows all ROIs to be transformed simultaneously.

In addition, the bit-mask representation also reduces CPU–to–GPU data transfer. Instead of transferring the CT volume together with more than twenty one-hot ROI channels, only the CT volume and a single compact bit-mask are transferred per iteration. This reduces I/O overhead and improves training throughput. Once on the GPU, the bit-mask *B* is decoded into binary ROI masks as required. When forming the network input, the decoded ROI masks $\hat{S}\in \mathbb {R}^{N_{\textrm{ROI}}\times D\times H\times W}$ are concatenated with the CT volume to obtain

13 $$\begin{aligned} I_{\textrm{in}} \in \mathbb {R}^{(1+N_{\textrm{ROI}})\times D\times H\times W}. \end{aligned}$$

Thus, the network operates on standard one-hot ROI channels, while the bit-mask is used only as an intermediate encoding.

The decoding is implemented via lightweight, element-wise bit-test, as shown in Fig. 3. In particular, the binary mask for ROI$_i$ is obtained via

14 $$ \begin{aligned} S_i(\textbf{r}) = \textrm{bool}\!\left( B(\textbf{r})\; \& \;(1 \ll (i-1)) \right) , \end{aligned}$$

where $\textrm{bool}(\cdot )$ maps nonzero values to 1, “ &” denotes the bitwise AND, and “≪” denotes the bit left-shift operation. These operations are highly efficient on modern GPUs and introduce negligible computational overhead.

During loss computation, only the bit-mask *B* remains resident in GPU memory, and the binary mask for each ROI$_i$ is decoded on demand at the moment its corresponding metric $M^{\textrm{pred}}_{r,k}$ or $M^{\textrm{gt}}_{r,k}$ is evaluated. This on-demand decoding strategy avoids storing all $N_{\textrm{ROI}}$ ROI channels simultaneously and thereby reduces peak GPU memory usage.

## Experiments and results

### Experiment setup

The ROIs used in our experiments are listed in Table 2. The template contains two PTV structures ($\textrm{PTV}_{54.25}$ and $\textrm{PTV}_{70}$) and 28 OARs, resulting in a total of $N_{\textrm{ROI}} = 30$ structures. Because this number fits within a 32-bit range, all ROI masks are encoded using a 32-bit unsigned integer (uint32) following the bit-mask representation described in Section Bit-mask encoding.

For the CDM loss, all PTV-related $V_{95\%}$ metrics were included in the differentiable surrogate of *V-metrics*. To ensure controlled approximation accuracy, we adopt a margin of m=0.5 Gy (i.e., a 1 Gy window) and a tolerance of $\varepsilon =1\%$. Using the lower-bound condition in Eq. (8), we compute the minimum α values that satisfy the prescribed tolerance. Thus, we set α=209 for $\text {PTV}_{54.25}$ and α=176 for $\text {PTV}_{70}$. To reflect the higher clinical priority of PTVs, all PTV-related metric weights are set to w(r,k)=1, whereas all OAR-related metric weights are set to w(r,k)=0.1. The balancing coefficients are set to $\lambda _1 = 1$ and $\lambda _2 = 0.5$ for the final loss $\mathcal {L}_{\textrm{total}}$.

Unless otherwise specified, all experiments used a 3D U-Net baseline implemented in the MONAI^1^ framework. Models were trained for 1000 epochs using the AdamW optimizer with an initial learning rate of $3\times 10^{-4}$ and a cosine annealing schedule. Mixed-precision (bf16-mixed) training was employed for efficiency. All experiments were conducted on an NVIDIA RTX 6000 Ada GPU.

### Results

**Table 3:** Comparison of model performance under different loss function configurations. Lower scores indicate better performance

Loss function	PTV Score (%) ↓	OAR Score (Gy) ↓	Dose Score (Gy) ↓
MAE	1.544 ± 1.188	2.103 ± 0.704	**1.300 ± 0.229**
MAE + DVH	0.992 ± 0.466	2.162 ± 0.518	1.385 ± 0.217
MAE + DVH + CDM	0.567 ± 0.300	**1.933 ± 0.481**	1.389 ± 0.228
MAE + CDM (proposed)	**0.491 ± 0.250**	1.999 ± 0.497	1.370 ± 0.245

The best performance for each metric is highlighted in bold

**Fig. 4:**
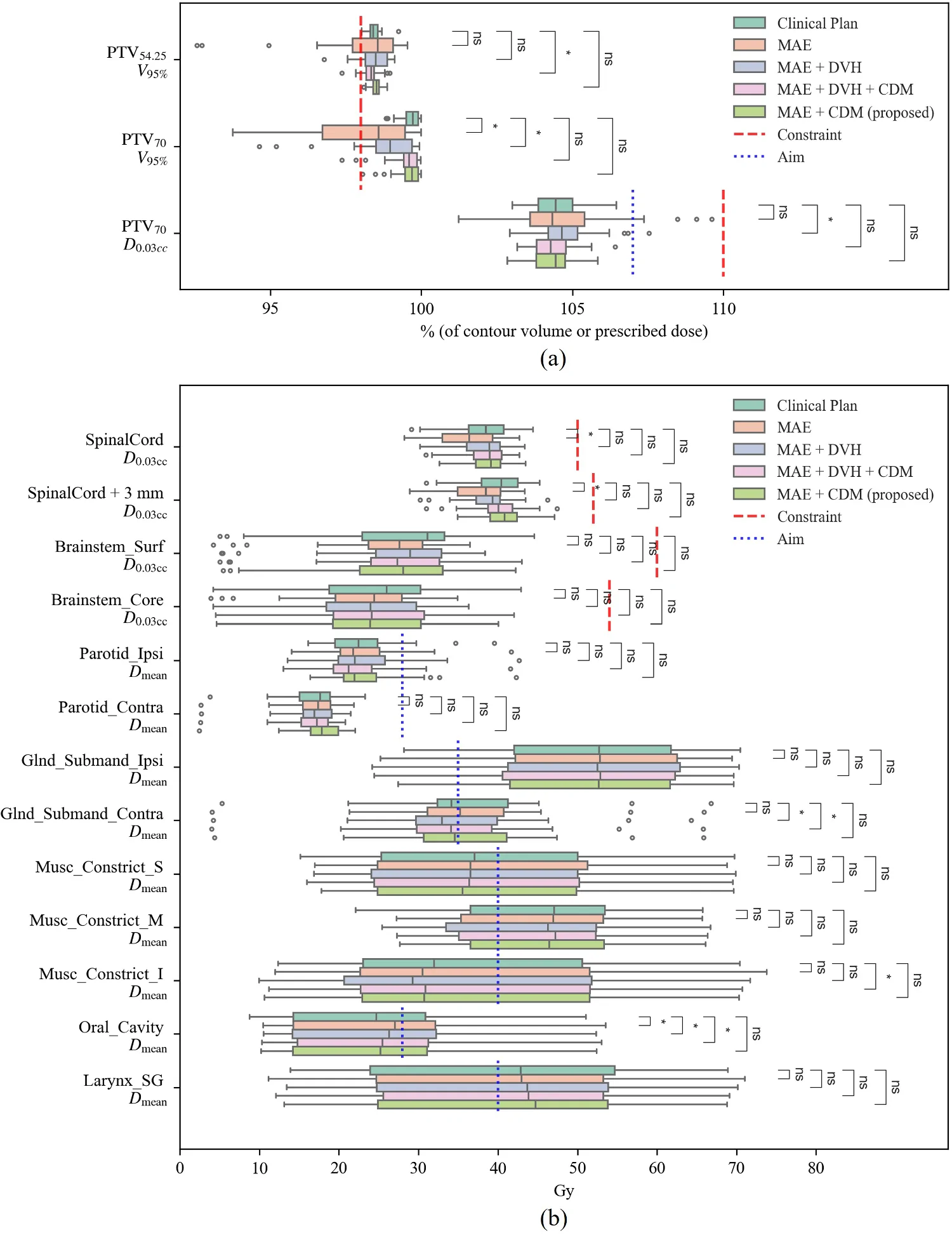
Boxplots of dose evaluation metrics for the clinically most relevant ROIs for different loss function configurations. **a** Boxplots for PTVs. **b** Boxplots for OARs. The corresponding planning aims and dose constraints are also shown (aims are clinically regularly violated due to proximity of the tumor). Parotid and submandibular glands were categorized as ipsilateral (Ipsi) or contralateral (Contra) according to received dose. Statistical significance was tested using a two-tailed Wilcoxon signed-rank test. *: p≤ 0.05, ns = not significant

**Fig. 5:**
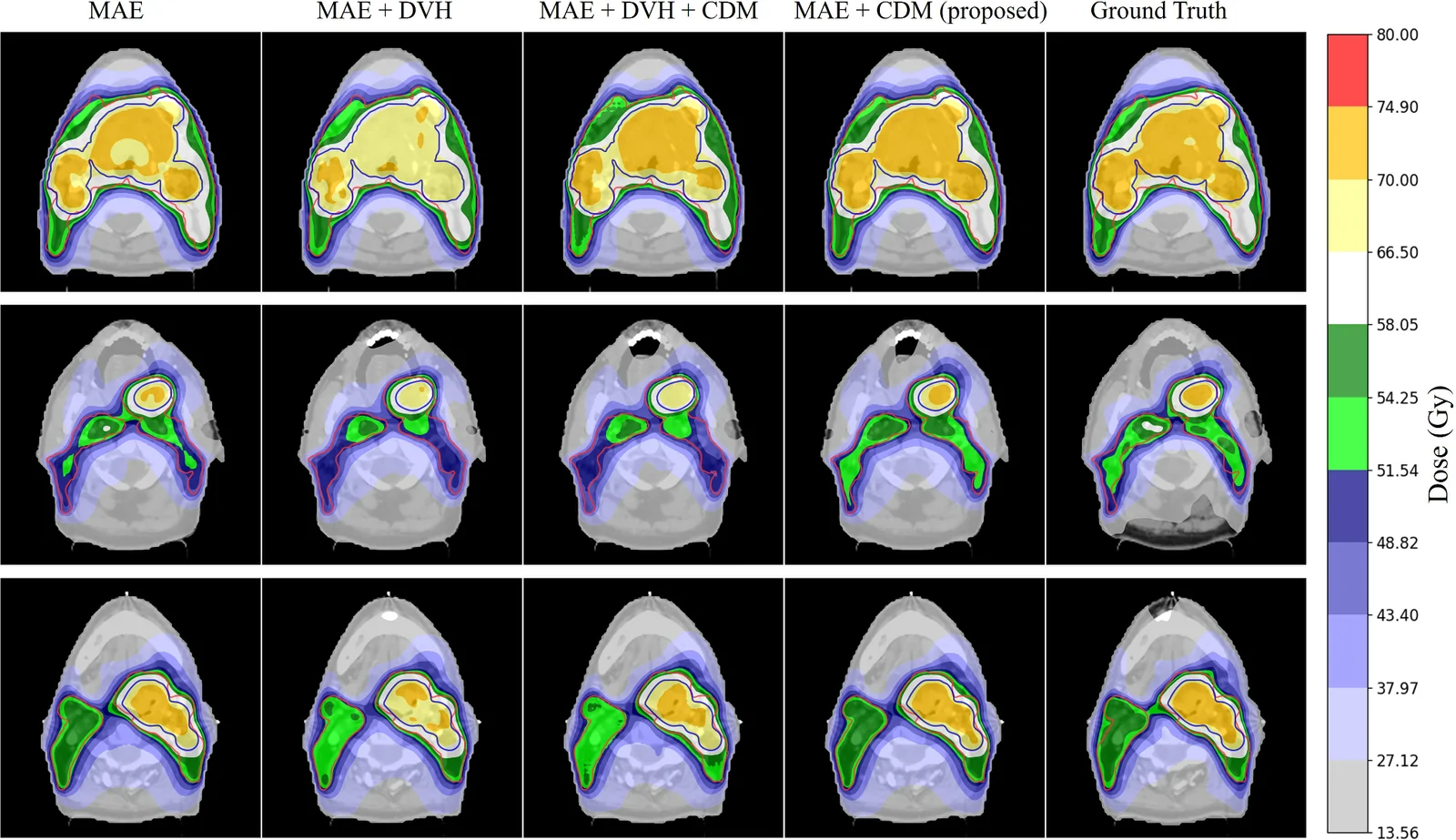
Axial visualization of dose distributions from three patients (one patient per row) under different loss function configurations. The red contour denotes the $\text {PTV}_{54.25}$, while the blue contour represents the $\text {PTV}_{70}$. The main feature to compare across different loss function configurations is the PTV coverage. A colormap routinely used in clinical practice is employed to enhance visual interpretability

#### Loss function comparison

We first compared different loss function configurations using the 3D U-Net baseline to evaluate the impact of the proposed CDM loss. Four loss function configurations were investigated: MAE, MAE + DVH, MAE + DVH + CDM, and MAE + CDM (our proposed setting). Here, the DVH loss refers to the DVH curve loss introduced in [10], which provides an efficient way to penalize DVH curve discrepancies. Table 3 reports the model performance under different loss function configurations. It shows that introducing the CDM loss led to a substantial improvement in the PTV Score, achieving the lowest value when combined with MAE. In contrast, both MAE and MAE + DVH alone failed to effectively improve the PTV Score. The CDM loss also provided moderate benefits for the OAR Score. Although the MAE-only configuration yielded the lowest Dose Score, it also had a very high PTV Score..

We further examined whether the predicted dose distributions satisfied the predefined planning constraints. As illustrated in Fig. 4, the MAE + CDM configuration was the only setting that met both the $V_{95\%}$ ($\ge 98\%$) and $D_{0.03 cc}$ ($\le 110\%$) constraints across all test cases. No statistically significant differences from the clinical plans were observed for these metrics. In contrast, the other loss configurations showed instances of insufficient PTV coverage or excessive dose, as shown in Fig. 5. The proposed MAE + CDM achieves PTV coverage that more closely matches the clinical plans. For OARs, all loss configurations satisfied the relevant clinical limits, but only the MAE + CDM model showed no statistically significant differences from the clinical plans for all metrics.

#### Ablation study

To assess the influence of α used in the differentiable *V-metric* approximation, we performed an ablation study on the parameter α (Table 4). Smaller values (e.g., 100) resulted in higher PTV Scores. The theoretically derived values (209, 176) for $\text {PTV}_{54.25}$ and $\text {PTV}_{70}$ achieved the best PTV Score while maintaining stable OAR and Dose Scores. Increasing α beyond this range (300–500) did not yield further improvement and in some cases slightly degraded PTV performance.

To further assess the computational efficiency of the proposed bit-mask ROI encoding, an ablation study was performed using the 3D U-Net baseline. The comparison is reported in Table 5. When using conventional one-hot ROI channels, training required a peak GPU memory of 20.50 GB and an average epoch duration of 241 s. With bit-mask encoding, the peak memory was 19.57 GB (4.5% reduction) and the epoch duration was 43 s (82.2% reduction). Peak GPU memory is reported because memory usage during training is dynamic, and the maximum allocation determines whether the model fits within available GPU memory.

**Table 4:** Ablation study on the parameter α. Lower values indicate better performance

$\alpha ~(\text {PTV}_{54.25})$	$\alpha ~(\text {PTV}_{70})$	PTV Score (%) ↓	OAR Score (Gy) ↓	Dose Score (Gy) ↓
100	100	0.564 ± 0.287	**1.919 ± 0.502**	1.349 ± 0.228
209	176	**0.491 ± 0.250**	1.999 ± 0.497	1.370 ± 0.245
300	300	0.500 ± 0.276	1.961 ± 0.491	**1.346 ± 0.226**
400	400	0.563 ± 0.298	1.988 ± 0.475	1.352 ± 0.214
500	500	0.563 ± 0.308	1.993 ± 0.452	1.327 ± 0.204

The best performance for each metric is highlighted in bold

**Table 5:** Ablation experiment on the 3D U-Net baseline comparing computational efficiency with and without the bit-mask ROI encoding

Method (3D U-Net baseline)	Peak GPU Memory (GB)	Epoch Duration (s)
Without bit-mask	20.50	241
With bit-mask (proposed)	**19**.**57**	**43**

The best performance for each metric is highlighted in bold

#### Model comparison

**Table 6:** Comparison of dose prediction performance across different network architectures using the MAE + CDM loss configuration. Lower scores indicate better performance

Model	PTV Score (%) ↓	OAR Score (Gy) ↓	Dose Score (Gy) ↓
SwinUNETR [13]	0.699 ± 0.292	2.035 ± 0.566	1.461 ± 0.253
C3D [4]	0.497 ± 0.314	**1.945 ± 0.473**	**1.289 ± 0.192**
MedNeXt [14]	0.496 ± 0.251	2.154 ± 0.571	1.395 ± 0.204
Pyfer [6]	**0.491 ± 0.287**	2.019 ± 0.517	1.290 ± 0.207
3D U-Net (baseline)	**0.491 ± 0.250**	1.999 ± 0.497	1.370 ± 0.245

The best performance for each metric is highlighted in bold

Building upon the best-performing loss setting (MAE + CDM), we finally compared the 3D U-Net baseline with several state-of-the-art architectures (Table 6), including transformer-based models (SwinUNETR [13]), large-kernel ConvNeXt-style convolutional networks (MedNeXt [14]), and cascade-style frameworks (C3D [4], Pyfer [6]). Notably, the 3D U-Net achieved a PTV Score of 0.491, matching the best-performing model (Pyfer) and outperforming more complex architectures such as SwinUNETR and MedNeXt. The OAR Score of 1.999 was also competitive, ranking second among all models.

## Discussion

Overall, our results demonstrate that directly optimizing clinically used dose metrics is substantially more effective than voxel-wise or DVH curve-based supervision. As shown in Table 3, MAE alone achieved the lowest voxel-wise Dose Score, but failed to ensure adequate target coverage. Previously proposed DVH-based losses improved DVH curve similarity [8, 9]. However, they did not reliably enforce clinically mandatory constraints, such as $V_{95\%}$ for PTVs. This limitation is evident in Fig. 4 and  5. Both MAE and MAE + DVH exhibit cases of insufficient target coverage or excessive hot spots. In contrast, the proposed CDM loss directly optimizes the dose metrics used in clinical plan evaluation. This task-aligned supervision consistently satisfied all PTV constraints in all patients, without compromising OAR sparing.

A key component of the proposed framework is the differentiable surrogate for *V-metrics*, which introduces a sigmoid slope parameter α that controls the trade-off between approximation accuracy and gradient stability. The analytically derived lower bound provides a principled way to select α without exhaustive empirical tuning. As shown in the ablation study (Table 4), overly small values of α resulted in insufficient discrimination around the dose threshold, while increasing α beyond the derived bound did not yield further improvement. Importantly, performance remained stable across a reasonable range of α values, indicating that the proposed selection should be interpreted as a conservative and robust choice rather than a finely tuned optimum.

An important advantage of the proposed CDM loss is its flexible, template-driven design. All optimized metrics, ROIs, and their relative priorities are specified through a simple JSON-based clinical evaluation template. This allows the loss to be configured for different planning protocols without modifying the network architecture or training pipeline. Although this study focuses on a single-institution H&N dataset, the template-based formulation in principle can be adapted to other datasets by updating the clinical criteria template. Compared with fixed, hard-coded loss formulations, this approach provides a practical and transparent way to align training objectives with clinically used evaluation standards.

An additional observation from the model comparison experiments is that architectural complexity becomes a secondary factor when the training objective is well aligned with clinical goals. Under the MAE + CDM loss, a standard 3D U-Net achieved performance comparable to, or better than, transformer-based [13] and cascade-style architectures [4, 6] (Table 6). This suggests that, for 3D dose prediction, improving *what the model is optimized for* may be more impactful than increasing architectural complexity. From a clinical deployment perspective, this is an encouraging result, as simpler architectures are typically easier to train, validate, and maintain.

Several limitations of this study should be acknowledged. First, all data were obtained from a single institution with a unified planning protocol. The generalizability of the approach to other institutions therefore remains to be validated. Second, while the predicted dose distributions satisfied all clinical constraints, this study did not evaluate downstream integration into deliverable treatment planning [1]. Future work will focus on multi-institutional validation and on assessing the impact of CDM-guided dose prediction on dose mimicking and final plan quality.

In summary, this work presents a clinically aligned framework for 3D H&N dose prediction by directly optimizing clinical DVH evaluation metrics and introducing an efficient, lossless ROI encoding strategy. The proposed approach consistently satisfies clinical constraints and improves the clinical acceptability of predicted dose distributions, providing a practical foundation for AI-assisted radiotherapy planning.

## Supplementary Information

Below is the link to the electronic supplementary material.

Supplementary file 1 (pdf 133 KB)

## Footnotes

1 https://github.com/Project-MONAI/MONAI.
