# Supplementary material for: Clinical DVH metrics as a loss function for 3D dose prediction in head and neck radiotherapy
Source: Int J Comput Assist Radiol Surg. 2026 Aug 13;21(11):2363–73. doi: 10.1007/s11548-026-03761-6 (PMC13759592; doi:10.1007/s11548-026-03761-6)
Supplement: Supplementary file 1 [file 11548_2026_3761_MOESM1_ESM.pdf]

1 Derivation of the α Bound for Differentiable *V–metrics*

This section provides the full derivation of the lower bound on the sigmoid slope parameter α used in the differentiable surrogate of *V–metrics*.

Let d_i denote the dose at voxel i within a region of interest (ROI), and let T be the dose threshold defining a given *V–metric*. We define

$$z_i = d_i - T. \tag{1}$$

The true *V–metric* is given by

$$V_x = \frac{1}{N} \sum_{i=1}^{N} H(z_i), \tag{2}$$

where $H(\cdot)$ denotes the Heaviside step function. Its differentiable approximation using a sigmoid with slope α is

$$V_x^{\text{approx}}(\alpha) = \frac{1}{N} \sum_{i=1}^{N} \sigma_\alpha(z_i), \qquad \sigma_\alpha(t) = \frac{1}{1 + e^{-\alpha t}}. \tag{3}$$

1.1 Bounding the Approximation Error

We first define the mean pointwise approximation error:

$$\delta(\alpha) = \frac{1}{N} \sum_{i=1}^{N} \left| H(z_i) - \sigma_\alpha(z_i) \right|. \tag{4}$$

By the triangle inequality, the error in the *V–metric* satisfies

$$\left| V_x - V_x^{\text{approx}}(\alpha) \right| \le \delta(\alpha). \tag{5}$$

Using the identity

$$|H(z) - \sigma_\alpha(z)| = \begin{cases} 1 - \sigma_\alpha(z) = \dfrac{1}{1 + e^{\alpha z}}, & z \ge 0, \\ \sigma_\alpha(z) = \dfrac{1}{1 + e^{-\alpha z}}, & z < 0, \end{cases} \tag{6}$$

we obtain the symmetric bound

$$|H(z) - \sigma_\alpha(z)| = \frac{1}{1 + e^{\alpha |z|}} \le e^{-\alpha |z|}. \tag{7}$$

Therefore,

$$\delta(\alpha) \leq \frac{1}{N} \sum_{i=1}^{N} e^{-\alpha |z_i|}. \tag{8}$$

1.2 Margin-based Error Decomposition

Let $m > 0$ define a symmetric margin around the threshold, and let

$$q_m = \frac{1}{N} \#\{\, i : |z_i| \leq m \,\} \tag{9}$$

denote the fraction of voxels whose dose values lie within this margin.

For voxels satisfying $|z_i| \leq m$, the worst-case approximation error is $1/2$, since $\sigma_\alpha(0) = 1/2$. For voxels with $|z_i| > m$, the error is bounded by $e^{-\alpha m}$. This yields

$$\delta(\alpha) \leq q_m \left(\tfrac{1}{2}\right) + (1 - q_m) e^{-\alpha m}. \tag{10}$$

1.3 Lower Bound on α

To ensure that the approximation error remains below a user-defined tolerance ε, it suffices to require

$$q_m \left(\tfrac{1}{2}\right) + (1 - q_m) e^{-\alpha m} \leq \varepsilon. \tag{11}$$

Solving for α yields the lower bound

$$\alpha \geq \frac{1}{m} \ln\left(\frac{1 - q_m}{\varepsilon - \frac{1}{2} q_m} \right), \tag{12}$$

which provides a sufficient (not necessarily tight) condition used in the main text to select the sigmoid slope parameter for each *V–metric*. In practice, the quantity q_m is estimated from the training set dose distributions, leading to the specific α values reported in Section 4.1 of the main manuscript.
